# A Functional Tricarboxylic Acid Cycle Operates during Growth of *Bordetella pertussis* on Amino Acid Mixtures as Sole Carbon Substrates

**DOI:** 10.1371/journal.pone.0145251

**Published:** 2015-12-18

**Authors:** Marie Izac, Dominique Garnier, Denis Speck, Nic D Lindley

**Affiliations:** 1 Université de Toulouse; INSA, UPSr, INP, LISBP, Toulouse, France; 2 INRA, UMR792 Ingénierie des Systèmes Biologiques et des Procédés, Toulouse, France; 3 CNRS, UMR5504, Toulouse, France; 4 Sanofi Pasteur, Marcy-l'Étoile, France; Laurentian University, CANADA

## Abstract

It has been claimed that citrate synthase, aconitase and isocitrate dehydrogenase activities are non-functional in *Bordetella pertussis* and that this might explain why this bacterium’s growth is sometimes associated with accumulation of polyhydroxybutyrate (PHB) and/or free fatty acids. However, the sequenced genome includes the entire citric acid pathway genes. Furthermore, these genes were expressed and the corresponding enzyme activities detected at high levels for the pathway when grown on a defined medium imitating the amino acid content of complex media often used for growth of this pathogenic microorganism. In addition, no significant PHB or fatty acids could be detected. Analysis of the carbon balance and stoichiometric flux analysis based on specific rates of amino acid consumption, and estimated biomass requirements coherent with the observed growth rate, clearly indicate that a fully functional tricarboxylic acid cycle operates in contrast to previous reports.

## Introduction

The bacterium *Bordetella pertussis* is the causative agent of whooping cough. This disease was responsible for high world-wide mortality prior to development of vaccination strategies, particularly among children. Implementation of high-coverage vaccination from the first months of life of an infant has been the solution to decrease its incidence [1–3]. However, over the past ten years, renewed outbreaks in whooping cough cases have appeared worldwide. The disease therefore remains a major priority for pharmaceutical companies [1,4,5]. In the 1940s, whole-cell vaccines were first manufactured with inactivated cells. While effective in stimulating the immune response, cellular compounds such as lipopolysaccharides (LPS) induced non-negligible and even life-threatening side effects [3]. In the 1980s, the first acellular vaccines were developed, including the inactivated form of pertussis toxin, the major antigen of *B*. *pertussis*. Nowadays, acellular vaccines have evolved to contain at least two and up to five antigens, depending on the countries and manufacturers [1,2].

In all cases, manufacture of whole-cell or acellular vaccines is directly linked to the capacity to cultivate *B*. *pertussis*. Indeed, whole-cell vaccines are obtained from harvested cells of industrial scale cultures, whereas some antigens for acellular vaccines are either recovered from culture supernatant or from cells, depending on the vaccine formulation. Increased knowledge of the physiological determinants which condition growth response of *B*. *pertussis* is therefore an important challenge to facilitate efficient and safe production of vaccines. While culture media have been developed to attain satisfactory growth, the underlying understanding of metabolic aspects of growth has not received much attention. The completed genome sequence enables potential metabolic topology to be predicted though it should be noted that the genome appears still to be evolving [6,7]. Some of our understanding of how central metabolism is thought to function pre-dates the sequence and needs to be re-examined in light of this additional data.

In accordance with growth experiments, sequencing analysis confirms that *B*. *pertussis*, like some phylogenetically similar bacteria, cannot grow on classical carbohydrates due to an incomplete glycolysis pathway [8,9]. Two compulsory enzymes are not encoded: glucokinase and phosphofructokinase, as well as an essential part of the PTS transport system [9]. Gluconeogenesis is however fully functional thereby supplying the various anabolic growth precursors during growth on non-sugar substrates. The pentose phosphate pathway is only functional regarding the non-oxidative branch as glucose-6-phosphate dehydrogenase and 6-phosphogluconate dehydrogenase enzymes are absent [9]. The Tricarboxylic acid (TCA) cycle enzymes are all theoretically encoded in *B*. *pertussis* genome though it has been claimed that this pathway is only partially functional, three enzymes being considered to be functionally absent: citrate synthase, aconitase and isocitrate dehydrogenase [10,11]. Carbon substrates are rather limited and growth experiments have shown that only glutamic acid, proline and α-ketoglutarate are used by *B*. *pertussis* to sustain growth. All these substrates share a common catabolic pathway at the α-ketoglutaric acid level. Moreover, growth is arrested as soon as these compounds are depleted in the liquid medium, despite the presence of other potential carbon sources [10,11].

Amino acid biosynthesis pathways are generally not impaired though growth is improved in complex media [12,13]. Since oxaloacetate is one of the precursors for the aspartate family of amino acids, the TCA cycle must be functional between α-ketoglutaric acid and oxaloacetate. Further metabolism of TCA cycle C4-dicarboxylic acids (malate and/or oxaloacetate) into gluconeogenesis at the level of PEP/pyruvate is necessary to feed central metabolic pathways upstream of pyruvate and the acetyl-CoA for fatty acid biosynthesis. This decarboxylation step to couple oxidative TCA cycle to gluconeogenesis is covered by the malic enzyme [9]. The doubt existing over the presence of a fully functional TCA cycle is supported principally by the observations that citric acid cannot replace the other major carbon substrates and by frequent observations that *B*. *pertussis* has a documented capacity to accumulate free fatty acids and/or polyhydroxybutyrate (PHB) under certain conditions (both being possible acetyl-CoA overflow metabolites) [10,14]. However, a recent study on the modeling of fed-batch culture strategies has suggested that this hypothesis is possibly incorrect, based upon gas transfer kinetics (oxygen consumption and carbon dioxide emission rates) [15]. To formulate optimized fermentation strategies, it is essential that this aspect of the central metabolic pathways can be fully understood. Full carbon stoichiometric flux analysis coupled to gene expression analysis and biochemical analysis of the three enzyme activities necessary to ensure a fully functional TCA cycle were examined to resolve this aspect of the physiology of *B*. *pertussis* using the sequenced Tohama I strain. These approaches indicate clearly that a fully functional TCA cycle operates in *B*. *pertussis*.

## Materials and Methods

### Strains

*B*. *pertussis* strain Tohama I from the ATCC (BAA-589) was used throughout this study (http://www.lgcstandards-atcc.org). The genome of this strain has been sequenced [9].

*Escherichia coli* K-12 MG1655 was used for enzymatic assays [16,17].

### Culture media

Bordet-Gengou agar was used to initiate *B*. *pertussis* strain Tohama I growth (BD Diagnostic Systems, ref. 248200). Agar was enriched with 20% defibrinated sheep’s blood (Oxoid, ThermoFisher, ref. SR0051C). Liquid medium used in this study was derived from the Stainer and Scholte chemically defined medium [18]. Free L-amino acids (including glutamate and proline) at concentrations equivalent to those of complex casamino acid media were supplemented into the medium to enable precise amino acid consumption rates to be measured for carbon balancing and flux analysis. Beta-cyclodextrin and methylcellulose have also been added as these compounds have been reported to have positive effects on growth [19–21] while sodium chloride and Tris buffer concentrations have been diminished as compared to the original medium composition since pH was regulated. Concentrations per liter of liquid medium were: monosodium glutamate (10.9 g), proline (1.4 g), NaCl (0.6 g), Tris-base (1.5 g), beta-cyclodextrin (1.7 g) and methylcellulose (0.1 g). Foaming was diminished by adding 300 μl.l^-1^ of pure antifoam C (a 30% emulsion of silicone, Dow Corning). The medium was heat-sterilized at 121°C for 30 minutes. A concentrated growth factor solution included thermolabile compounds was prepared as previously described [12,14,22], filter sterilized (Minisart polyamide 0.2 μm, Sartorius, Göttingen, Germany) and added to sterile liquid medium just before inoculation with a 1% (v/v) ratio.

### Growth conditions

Lyophilized pellets from freeze-dried vials were rehydrated with 1 ml of the liquid culture medium and transferred into 50-ml shake flasks containing 10 ml of liquid culture medium. Rehydration was carried out for one hour at 36°C and under 125 rpm of agitation. 4 ml of suspension was then used to inoculate three Bordet-Gengou agar plates. Plates were incubated at 36°C for 72 hours with a non-inoculated control plate. Three 500-ml shake flasks with 100 ml of liquid medium and 1% (v/v) of growth factor solution were inoculated from the harvested bacterial suspension. The first liquid precultures were incubated at 36°C under a 200 rpm rotary shaker until the OD_650nm_ reached 0.8–1.0. This bacterial suspension was used to inoculate 3 1-liter shake flasks with 200 ml of liquid medium and 1% (v/v) of growth factor solution with a targeted initial OD of 0.07–0.08. Second precultures were incubated at 36°C under 200 rpm agitation until OD_650nm_ again reached 0.8–1.0. These precultures were then used to inoculate three independent 2-liter Biostat B^®^ Plus fermenters (Sartorius) containing 1.60 l liquid medium and 1% (v/v) of growth factor solution with an initial OD_650nm_ of 0.08. Temperature was controlled throughout growth at 36°C, pH was maintained at 7.3 with automatic addition of 5N *ortho*-phosphoric acid and dissolved oxygen (DO) concentration was controlled at 25% saturation with a constant air flow of 0.2 l.min^-1^ and agitation rate varying from 150 to 800 rpm to maintain the DO set point. Since antifoam was added before sterilization, no further additions were necessary during growth.

### Biomass determination

The optical density (OD) of the bacterial suspension was measured at 650 nm to follow growth. These values were transformed to dry cell weight (DCW) values using a pre-determined and experimentally confirmed ratio of 0.55 g_DCW_/unit of DO_650nm_ which was validated to hold true throughout the entire growth curve.

### Amino acid quantification

Free amino acid concentrations in 0.22 μm-filtered supernatants were measured by HPLC as follows. Proteins in the samples were precipitated by adding 4 volumes of pure methanol to 1 volume of the sample and incubating the mixture overnight at 4°C. Norvaline was included as the internal standard with a final concentration of 15 mM. The mixture was then centrifuged, and supernatants were kept for amino acid analysis as previously described [23]. Amino acids were automatically derived with *ortho*-phthalic aldehyde (OPA) and 9-fluorenylmethyl chloroformate (FMOC). The derivatives were separated on a Hypersil AA octadecylsilane column (reversed-phase HPLC, Agilent Technologies, Waldbronn, Germany) at 40°C in acetate buffer (pH 7.2) with triethylamine (0.018%) and tetrahydrofurane (0.03%). A linear gradient was used to increase the hydrophobicity of the mobile phase up to 60% acetonitrile. A diode array detector was used at 338 nm for OPA derivatives and at 262 nm for FMOC derivatives. These analyses were performed in duplicate on three biologically independent batches.

### Gas analysis

Oxygen and carbon dioxide concentrations in effluent gas were monitored on-line with a 3000 micro GC from SRA Instruments. The carrier gas was helium (Air Liquid). Two modules were used: an initial Molsieve 5A Plot column facilitating O_2_ and N_2_ detection and separation, and a second Plot U column module for CO_2_ detection. A filter was placed before the injection loop to obtain a dry and contaminant-free sample. Calibration for O_2_, N_2_ and CO_2_ was done with the compressed air used in fermentation, and a commercial standard with 85% N_2_, 10% O_2_ and 5% CO_2_ (Air Liquid).

### RNA extraction and cDNA synthesis

Triplicate sampling was undertaken at three distinct times in the culture: 8 hours, 16 hours and 24 hours, from the three biologically independent batch cultures. Samples were immediately frozen in liquid nitrogen and kept at -80°C until use. After thawing on ice, 4 mg of cells from each sample were broken at 4°C with a FastPrep-24 instrument (MP Biomedicals, Illkirch, France) with two cycles of 30 sec (maximal speed), interspaced with a 1-min cooling period. After centrifugation (4 min at 13,000 rpm and 4°C with an Eppendorf 5415R centrifuge), total RNAs from supernatants were extracted using an RNeasy^®^ Protect Bacteria Mini Kit (Qiagen). RNAs were quantified at 260 nm on a NanoDrop (Thermo Scientific), and their purity was assessed and controlled with 260/280 and 260/230 ratios above 2.00. The extracted RNAs were subjected to a DNase treatment with RNase-Free DNase (Qiagen) to remove residual genomic DNA. The purified RNA purity was controlled again with a NanoDrop with 260/280 and 260/230 ratios above 2.00. Moreover, Bionalyzer analysis were carried out on the purified RNAs that confirmed the absence of contamination by residual genomic DNA and the absence of degradation of the RNAs. For cDNA synthesis, 3μg of total purified RNAs were subjected to reverse transcription using the Superscript II reverse transcriptase (Life Technology). In addition, negative controls samples with no reverse transcribed mRNA were analyzed to confirm the absence of genomic DNA contamination.

### qPCR

Gene expression levels were analyzed using qPCR in micro-Fluidigm. The qPCR EvaGreen experiment was performed on a Biomark 96.96 dynamic array (Fluidigm, San Fransisco, California, USA). Among the samples and primers assessed with the chip, 4 pairs of primers (Table 1) were tested against 12 samples, corresponding to the three distinct times of culture (8h, 16h, 24h) for three biological replicates with a technical replicate for each sampling time. An internal control of human DNA was used to check efficiency and quality of the run. For each cDNA sample, targeted cDNA was amplified using the pool of primers and TaqMan^®^ PreAmp Master Mix (Fluidigm, San Francisco, California, USA) with the following program: (i) 10 minutes at 95°C, (ii) 14 cycles of 15 seconds at 95°C and 4 minutes at 60°C. The samples were then treated with exonuclease Exo I (for 30 minutes at 37°C for digestion and for 15 minutes at 80°C for inactivation). Finally, samples were added to a pre-mix (2X TaqMan Gene Expression Master Mix, 20X DNA Binding Dye Sample Loading Reagent, 20X EvaGreen^®^ and TE buffer) before being loaded into the macro-array. The sets of primers were loaded into the macro-array at a concentration of 20 μM. Control idnT values were used to normalize data.

**Table 1:** Primer pairs for qPCR analysis (F: foward primer; R: reverse primer).

Gene name (systematic name)	qPCR primer name	qPCR primer sequence (5' to 3')
*recA* (BP2546)	recA-F	TCATCGCCGAAATGCAGAAG
*recA* (BP2546)	recA-R	CTTCATGCGGATCTGGTTGA
*gltA* (BP2358)	gltA-F	ATCACACGATGGTCAACGAG
*gltA* (BP2358)	gltA-R	CTTGTACTCTTCACACGGGG
*acnA* (BP2014)	acnA-F	TGTCAGTTCTATTCGCTGCC
*acnA* (BP2014)	acnA-R	CAGCTTCATGTTCAGGTCGA
*acnB* (BP2021)	acnB-F	CCAAGCAAGGCAAGAAGAAC
*acnB* (BP2021)	acnB-R	CTCGTCTTCATAGCCGTTGG
*icd* (BP2488)	icd-F	TCATGAAGTTCACGGAAGGC
*icd* (BP2488)	icd-R	GAGATGTAGTCGCCGTTCAG

### Enzymatic assays

All the following steps were achieved at 4°C. *B*. *pertussis* Tohama I was grown in the chemically defined medium previously described. *E*. *coli* strain K-12 MG1655 was grown in minimal medium M9, supplemented with glucose and thiamine [17]. 20 mg of actively growing cells (OD_650nm_ = 1.0) were washed twice with a 0.2% KCl solution. The washed cell pellet was then resuspended in 1 ml of a Tris-carballylic acid buffer (Tris-tricarballylate pH 7.8, 2.7 M glycerol, 50 mM MgCl_2_, 300 mM DTT), used for cell disruption. Cells were lysed using a FastPrep-24 instrument (MP Biomedicals, Illkirch, France) and were subjected to six cycles of 30 sec (maximal speed), interspaced with 1-min cooling periods. After centrifugation (15 min at 13,200 rpm at 4°C), the cell-free crude extracts were maintained at 4°C prior to the assay procedures.

Enzymatic reactions were assessed with a SpectramaxPlus384 (Molecular Devices), thermostatically controlled at 36°C. All the reagents used were purchased from Sigma Aldricht (USA). They were mixed into a 96-well microplate, with a final volume of 200μl. In these conditions, the light path was equal to 0.75 cm. The optical density was measured at 7 sec intervals for 10 min with a 3 sec shaking period between each reading. Enzyme activities were determined from initial linear reaction phases. Only those crude extract concentrations showing linear relationship between reaction rate and cell extract dilution factor were retained for calculating specific activities. Reaction rates were expressed relative to total protein concentrations as quantified by Bradford assay (Bio-Rad Protein assay, Bio-Rad, Germany) at 595 nm, to obtain specific activities (μmol/min/mg_P_) [24]. Assays were done in duplicate for each biological triplicate in a range of dilutions.

Citrate synthase activity was assayed as described by Lapujade *et al*. (1998) [25] in a protocol adapted from Weitzman *et al*. (1969) [26] at 412 nm using DTNB (5,5′-Dithiobis(2-nitrobenzoic acid), molar extinction coefficient for DTNB of 13,600 μmol^-1^.ml^+1^.cm^-1^) as coupling reaction to measure coenzyme A (CoASH) liberation in the following reaction mixture (Tris-HCl buffer 500 mM pH 7.2, 37 μl; MgCl_2_ 50 mM, 20μl; acetyl-CoA 2 mM, 10 μl, DTNB 5 mM, 20 μl; oxaloacetate 6.5 mM, 5 μl and cell-crude extract, 108μl).

Isocitrate dehydrogenase activity was assayed as described by Lapujade *et al*. (1998) [25] in a reaction mixture containing Tris-HCl buffer 500 mM pH 7.2, 40 μl; MnSO_4_ 50 mM, 20 μl; Isocitrate 100 mM, 20 μl; NADP^+^ 6 mM, 20 μl and cell-crude extract 100 μl. The formation of NADPH was followed at 340 nm using the molecular extinction coefficient of 6,220 μmol^-1^.ml^+1^.cm^-1^.

Aconitase activity was assayed as described by Lapujade *et al*. (1998) [25] in a reaction mixture containing Tris-HCl buffer 500 mM pH 7.2, 38 μl; NaCl 500 mM, 20 μl; MnSO_4_ 50 mM, 20 μl; cis-aconitate 10 mM, 40 μl, NADP^+^ 6 mM, 20 μl, commercial (Sigma, ref. 94596) dehydrogenase 600 U/ml, 2 μl and cell-crude extract, 40 μl. NADPH formation was followed at 340 nm.

In all cases, control assays were undertaken to check for possible artefacts in mixtures lacking cell-crude extract, the triggering substrate or reaction cofactors.

### Carbon stoichiometric flux analysis

Carbon flux models were built using experimental data concerning specific rates of individual amino acid consumption, growth and gas transfer (oxygen uptake and CO_2_ production) from batch cultivations of *B*. *pertussis* Tohama I in which two periods of growth have been distinguished: the exponential growth period between 0–12 hours and a second period of slower growth from 12 hours-end of culture. The analysis therefore considers global quantities either consumed or produced on both periods, instead of consumption or production rates thereby diminishing risk of experimental errors. The model of central metabolism used takes into account pathways as reconstructed from genome–based pathway analysis as depicted in the Kyoto Encyclopedia of Genes and Genomes (KEGG; *Bordetella pertussis* Tohama I; T number: T00139, Org. code: bpe) and consists of the gluconeogenesis pathway, the TCA cycle (either fully or partially functional), the pentose phosphate pathway as well as amino acid biosynthesis/degradation pathways. KEGG pathway database for this microorganism comes directly from genome sequencing and annotation reported by Parkhill *et al*. (2003) [9] and levels of general homology with functional proteins seen in other bacteria have been confirmed here.

Outputs of the model for growth used central metabolic precursors as measured in phylogenetically similar bacteria *Cupriavidus metallidurans*, as determined by Ampe *et al*. (1997) [27]. Intermediate metabolites and energy requirements were expressed as the amount needed for the formation of one gram of biomass in exponential growth (Table 2). The specific amino acid content of *B*. *pertussis* Tohama I was determined after acid hydrolysis of biomass samples and was very close to that seen for *C*. *metallidurans*. The amino acid composition (expressed as micromoles per gram of cells) was as follows: Ala, 874; Arg, 339; Asp/Asn, 623; Cys, 19; Glu/Gln, 686; Gly, 680; His, 88; Ile, 228; Leu, 576; Lys, 287; Met, 125; Phe, 193; Pro, 269; Ser, 299;Thr, 352; Tyr, 141; and Val, 430. Regarding cofactor and energy requirements, the approach used for *C*. *metallidurans* involved calculating carbon flux throughout central metabolism during growth on a single carbon substrate, whereas *B*. *pertussis* metabolism relies on co-consumption of various amino acids. This aspect has therefore been accounted for in model estimations since several amino acids are provided in the culture medium and do not need to be synthesized, leading to a diminished energetic requirement.

**Table 2:** Intermediate metabolites and energy requirements for the formation of 1 g of biomass during exponential growth [27].

Precursor metabolite	Amount required for 1 g of biomass (μmol/g)
glucose-6-phosphate (G6P)	359
fructose-6-phosphate (F6P)	101
ribose-5-phosphate (R5P)	524
erythrose-4-phosphate (E4P)	338
glyceraldehyde-3-phosphate (GAP)	87
3-phosphoglycerate (3PG)	1,399
phosphoenolpyruvate (PEP)	655
pyruvate (PYR)	2,603
acetyl-CoA (AcCoA)	2,522
α-ketoglutarate (αKG)	1,623
oxaloacetate (OAA)	1,759
~P	40,789
NADH2	-2,888
NADPH2	18,333

## Results

### Macrophenotypic parameters of batch cultures of *B*. *pertussis* strain Tohama I

Three independent batch cultures of *B*. *pertussis* strain Tohama I were followed to characterize growth on the defined medium described above, based on commonly employed medium of Stainer & Scholte [18]. Growth characteristics were identical to those obtained using complex medium but analysis of specific amino acid consumption was simplified facilitating carbon recovery analysis.

**Fig 1:**
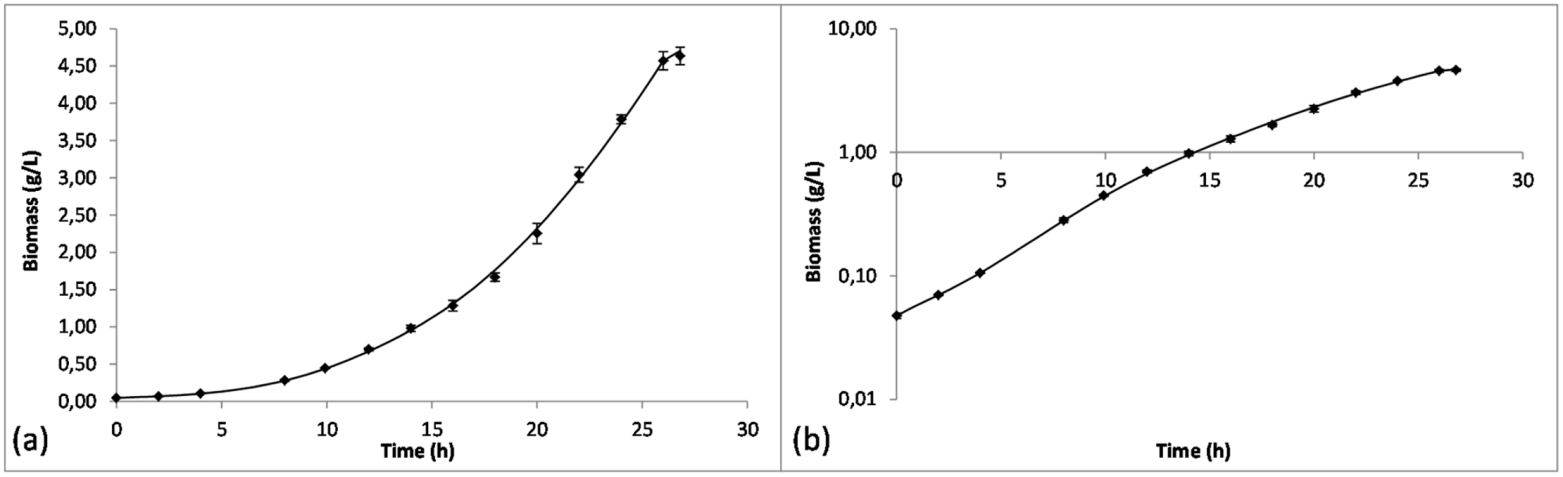
Kinetics of growth of *B*. *pertussis* Tohama I in batch cultures. Results are displayed as mean values for the three biological replicates with a regular Y-scale (a) and a logarithmic Y-scale (b). Error bars represent the replicates' standard deviations. Dry cell weight concentrations have been calculated from OD measurements at 650 nm.

**Fig 2:**
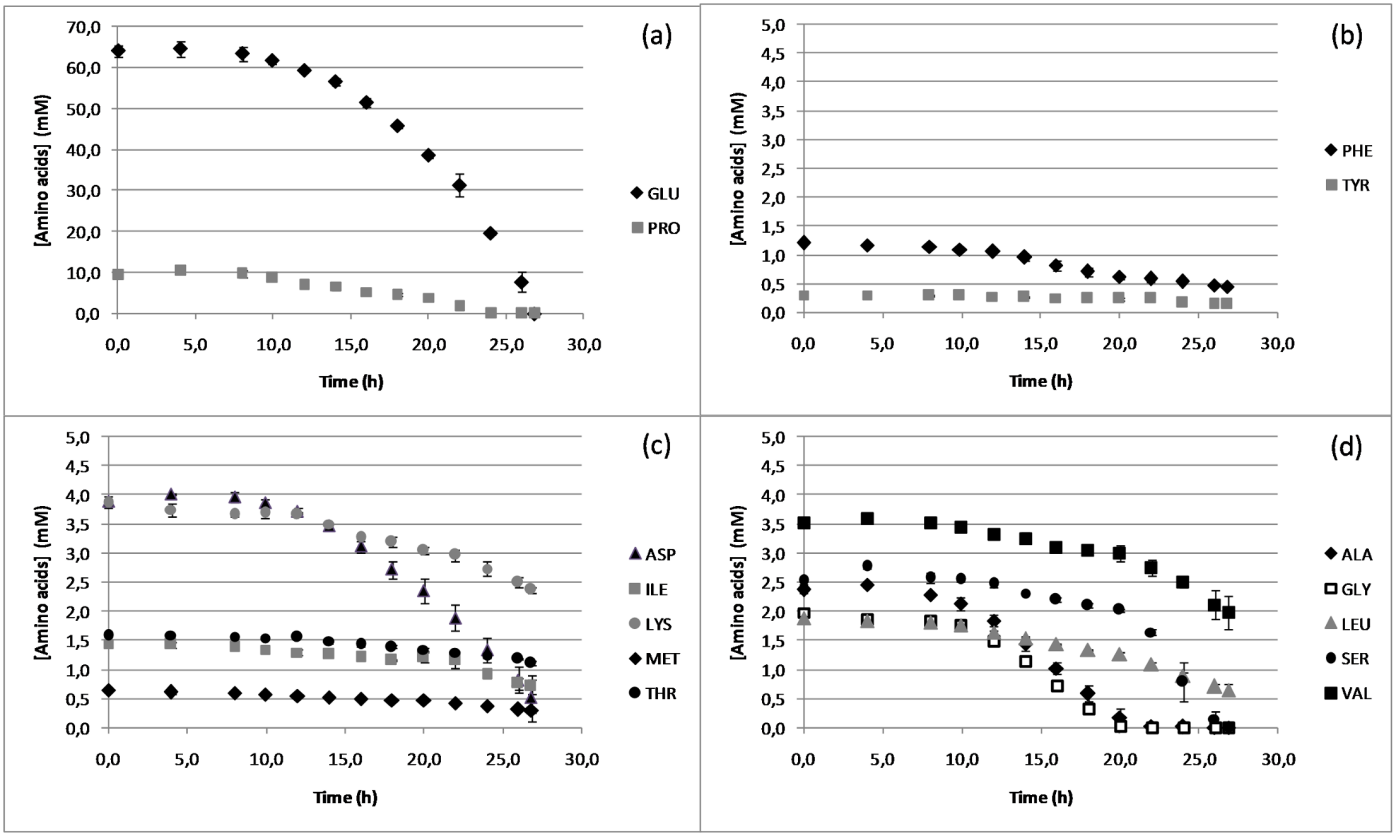
Kinetics of amino acid consumption of *B*. *pertussis* Tohama I in batches cultures. (a): Kinetics of glutamate and proline; (b) kinetics of aromatic amino acids; (c): kinetics of amino acids derived from pyruvate; (d): kinetics of amino acids derived from oxaloacetate. Results are displayed as mean values for the three biological replicates and error bars represent standard deviations.

An initial exponential growth phase was obtained (Fig 1) over the first 12 hours of growth (μ = 0.230±0.002 h^-1^) while growth rate decreased in the second part of the culture (μ = 0.130±0.001 h^-1^) concomitant with the modification of the amino acid content of the growth medium (Fig 2). The end of the culture was detected when O_2_ consumption ceased and was linked with the glutamate depletion, the main carbon source of the microorganism. Proline, alongside aspartic acid, serine, alanine and glycine were all depleted before the end of culture, whereas residual amounts of other amino acids were detected after growth had stopped.

### Genes encoding for citrate synthase, aconitase and isocitrate dehydrogenase are present and expressed by *B*. *pertussis* Tohama I

The Sanger Institute sequenced the whole *B*. *pertussis* Tohama I genome with funding from the Wellcome Trust. It has been submitted to EMBL/GenBank with the accession numbers: BX470248 (Parkhill *et al*., 2003) [9]. The annotation revealed that orthologs of all the genes encoding the enzymes necessary for a functional TCA cycle are present in *B*. *pertussis* Tohama I genome (Table 3).

**Table 3:** Genes encoding Tricarboxicilic Acid Cycle enzymes in the *B*. *pertussis* Tohama I genome.

TCA cycle reaction	Gene name	Systematic name	Encoded Protein
pyruvate → acetyl-CoA	*aceE*	BP0993	pyruvate dehydrogenase component E1
pyruvate → acetyl-CoA	*aceE*	BP1121	
pyruvate → acetyl-CoA	*aceF*	BP0994	pyruvate dehydrogenase complex dihydrolipoamide-acetyltransferase
pyruvate → acetyl-CoA	*odhL*	BP1126	2-oxoglutarate dehydrogenase complex subunit dihydrolipoamide dehydrogenase
pyruvate → acetyl-CoA	*lpdA*	BP0995lpd	dihydrolipoamide dehydrogenase
pyruvate → acetyl-CoA	*lpdA*	BP0618lpd	
pyruvate → acetyl-CoA	*pdhA*	BP0629	pyruvate dehydrogenase component E1 subunitα
**acetyl-CoA+ oxaloacetate→ citrate**	***gltA***	**BP2358**	**type II citrate synthase**
**citrate →isocitrate (intermediary: *cis*-aconitate)**	***acnA***	**BP2014**	**aconitate hydratase**
**citrate →isocitrate (intermediary: *cis*-aconitate)**	***acnB***	**BP2021**	**bifunctional aconitate hydratase 2**
**isocitrate→ α-ketoglutarate**	***icd***	**BP2488**	**isocitrate dehydrogenase**
α-ketoglutarate→ succinyl-CoA	*odhB*	BP1125	dihydrolipoamide succinyltransferase
α-ketoglutarate→ succinyl-CoA	*sucA*	BP1124	2-oxoglutarate dehydrogenase
α-ketoglutarate→ succinyl-CoA	*odhL*	BP1126	2-oxoglutarate dehydrogenase complex subunit dihydrolipoamide dehydrogenase
α-ketoglutarate→ succinyl-CoA	*lpdA*	BP0995lpd	dihydrolipoamide dehydrogenase
α-ketoglutarate→ succinyl-CoA	*lpdA*	BP0618lpd	
succinyl-CoA→succinate	*sucD*	BP2540	succinyl-CoA synthase subunit α
succinyl-CoA→succinate	*sucC*	BP2541	succinyl-CoA synthase subunit β
succinate→ fumarate	*sdhA*	BP2361	succinate dehydrogenase flavoprotein (FAD) subunit
	*sdhB*	BP2360	succinate dehydrogenase iron-sulfur subunit
succinate→ fumarate	*sdhC*	BP2363	succinate dehydrogenase cytochrome B subunit
	*sdhD*	BP2362	succinate dehydrogenase hydrophobic membrane anchorprotein
fumarate →malate	*fumC*	BP0248	fumarate hydratase
malate→ oxaloacetate	*mdh*	BP2365	malate dehydrogenase
malate→ pyruvate	*maeB*	BP1120	NADP-dependent malic enzyme
malate→ pyruvate	*maeB*	BP3456	
malate→ pyruvate	*maeB*	BP1064	

In particular, genes encoding for the type II citrate synthase, aconitase and isocitrate dehydrogenase were present. In some cases, additional genes with good homology were present, such as a second citrate synthase (hypothetical protein, BP1352) and an aconitate hydratase (2-methylisocitrate dehydratase, BP2369). However, for the purpose of this study, only the following genes which have been validated in the Genbank database were assessed (in bold in Table 3).

If the genes necessary for a putative functional TCA cycle are present in the *B*. *pertussis* genome, the possibility that one or more genes were not expressed needed to be confirmed. To verify this, quantitative PCR (qPCR) was undertaken at three distinct times of the cultures: 8 hours, 16 hours and 24 hours (OD_650nm_ ≈ 0.5, 2.5 and 6.5 respectively) and *recA* was used as reference gene. Critical thresholds for considered genes are presented in Table 4 indicating that the genes were indeed expressed.

**Table 4:** Critical threshold (Ct) values for the genes *recA*, *gltA*, *ancA*, *acnB* and *icd* for three distinct times in *B*. *pertussis* Tohama I batch cultures.

	Ct				
Culture time (h)	Ct *recA*	Ct *gltA*	Ct *acnA*	Ct *acnB*	Ct *icd*
**8**	10.7 ± 0.6	12.4 ± 0.5	11.7 ± 0.8	11.8 ± 0.7	12.4 ± 0.5
**16**	10.8 ± 1.1	12.0 ± 1.0	11.7 ± 1.4	11.6 ± 1.3	13.1 ± 1.0
**24**	11.0 ± 0.4	12.7 ± 0.5	12.0 ± 0.5	11.8 ± 0.4	12.6 ± 0.9

Results are displayed as mean ± standard deviation (SD) from three biological replicates and a technical replicate.

Citrate synthase and isocitrate dehydrogenase genes were shown (ΔCt method) to exhibit higher levels of expression compared to aconitase genes, but all genes were expressed throughout the cultures with fairly constant values.

### The corresponding proteins are efficiently synthesized with functional activity

The genes are present in the genome and appear to be efficiently expressed. It remains therefore to be ascertained if proteins have corresponding enzymatic activities. Using well established enzymatic activity assays validated in our laboratory for *Escherichia coli* extracts, it was shown that all three enzyme activities were present throughout the fermentation (Table 5). Indeed, citrate synthase activity was significantly higher than that assayed using glucose-grown *E*.*coli* strain K-12 MG1655 extracts. In all cases, it was confirmed that no assay artefacts were present and that measured activity was dependent upon the entire assay mixture.

**Table 5:** Enzyme specific activities of citrate synthase, aconitase and isocitrate dehydrogenase in crude extract of *B*. *pertussis* Tohama I and *E*. *coli* K-12 MG1655.

		Citrate synthase	Aconitase	Isocitrate dehydrogenase
**Enzyme specific activity (x10** ^**3**^ **μmol/min/mg protein)**	*B*. *pertussis* Tohama I	3.39±0.30	2.51±0.29	24.1±1.94
	*E*. *coli* K-12 MG1655	0.17±0.01	1.12±0.40	65.4±7.8

Results are displayed as mean ± standard deviation (SD) from three biological replicates and a technical replicate.

It can therefore be concluded that the three enzyme activities which complete the tricarboxylic acid cycle are indeed present and that the entire TCA cycle could be functional in *B*. *pertussis* Tohama I, depending upon the specific substrate–dependent pathway flux.

### Carbon stoichiometric flux analysis through central metabolism confirms the need for an entirely functional TCA cycle

**Fig 3:**
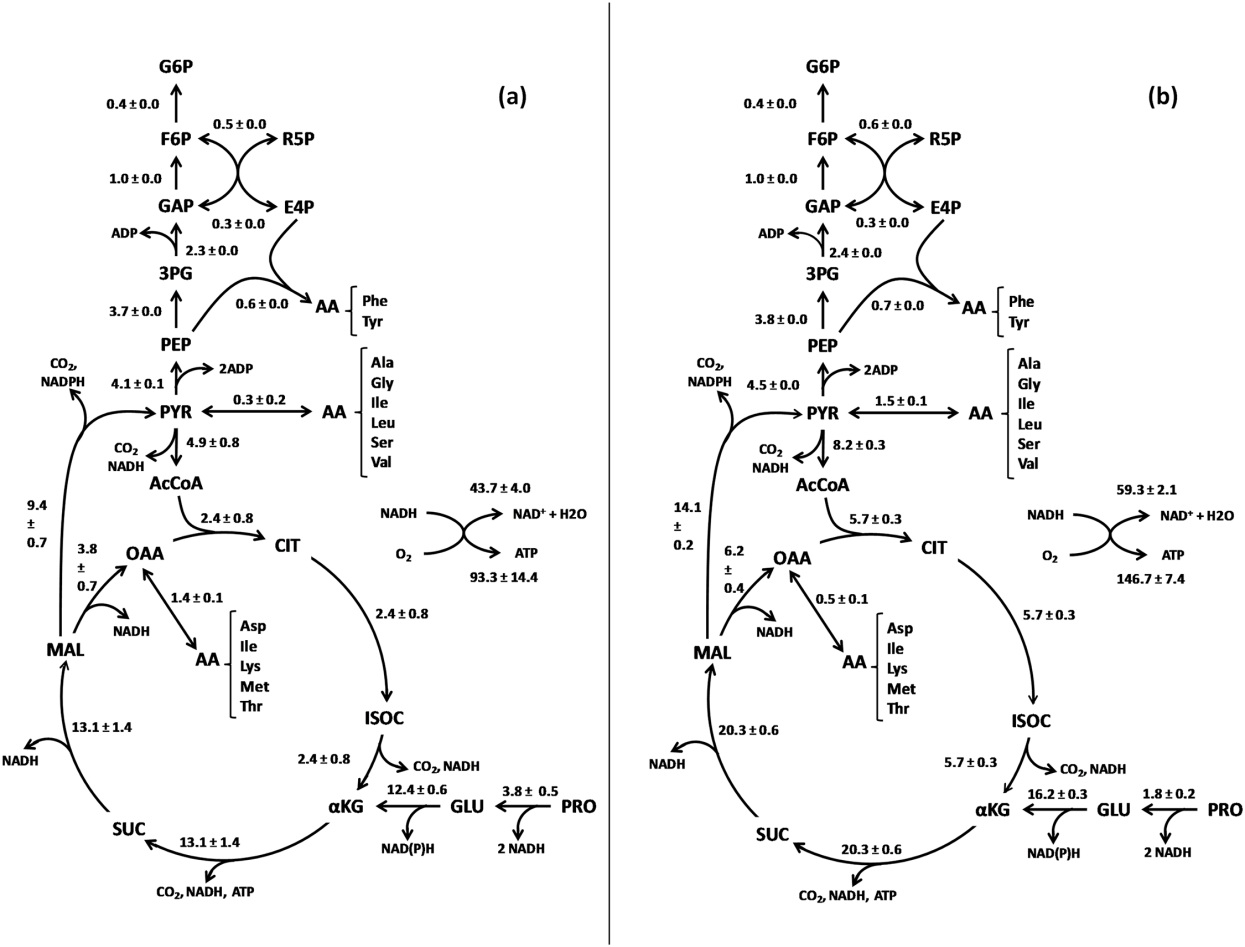
Estimation of carbon distribution within the central metabolic pathways based on the kinetic data from three biological replicates of *B*. *pertussis* Tohama I batch cultures. (a) Growth period: 0–12h; (b) Growth period: 12h-end of culture. G6P: glucose-6-phosphate; F6P: fructose-6-phosphate; R5P: ribulose-5-phosphate; E4P: erythrose-4-phosphate; GAP: glyceraldehyde-3-phosphate; 3PG: 3-phosphoglycerate; PEP: phosphoenolpyruvate; PYR: pyruvate; AcCoA: acetyl-CoA; CIT: citrate, ISOC: isocitrate; αKG: α-ketoglutarate; SUC: succinate; MAL: malate; OAA: oxaloacetate; GLU: glutamate; PRO: proline; AA: amino acids; Phe: phenylalanine, Tyr: tyrosine; Ala: alanine; Gly: glycine; Ile: isoleucine; Leu: leucine; Ser: serine; Val: valine; Asp: aspartate; Lys: lysine; Met: methionine; Thr: threonine. Numerical data are reported for the three replicates as mean ± standard deviation. They are expressed in mmoles per liter and per gram of biomass (mM/gX).

While different carbon labeling techniques can be employed to demonstrate that pathways are functional, a simpler method is to examine how substrate consumption data can be satisfied based upon known pathways encoded by the genome. This carbon flux analysis was based on experimental data generated from three independent batch cultures of *B*. *pertussis* Tohama I: amino acid consumption, growth and gas analysis (O_2_, CO_2_). No other soluble products were detectable and carbon recovery was satisfactory taking into account the inevitable analytical errors. Major carbon fluxes entering into *B*. *pertussis* central metabolism were linked to glutamate and proline consumption (Fig 3). They fuel the TCA cycle from α-ketoglutarate and allow carbon supply for the gluconeogenesis and the pentose phosphate pathways. At oxaloacetate and pyruvate levels, other consumed amino acids provide additional inputs for biomass precursor synthesis. Serine, aspartic acid and lysine needs are fully satisfied with their respective consumptions during the second growth period. Other amino acid requirements were guaranteed by their biosynthesis from central metabolites. Possible alternative pathways, involving lipid (other than those associated with cellular structures) or PHB synthesis were shown to be absent as these compounds were assessed to be close to the threshold detection limits of our analytical setup: <0.03 mM of free fatty acids in the extracellular medium and <7.0 x 10^−4^ gram of PHB per gram of biomass. Other commonly seen fermentation end-products were shown to be absent. Since amino acids are used as major carbon substrates, the amount of amino acids not directly incorporated into biomass has been checked by measuring ammonium production. The entire biomass precursor requirements can be satisfied without a functional TCA cycle but the flux which arrives at pyruvate cannot be absorbed in such a way as to generate the necessary amount of CO_2_ without a functional TCA cycle.

Taking into account the partial amino acid input into biomass via direct incorporation, energetic cofactor requirements were calculated to be somewhat lower than those normally used for such estimations. The carbon flux estimations show that only malic enzyme generated directly the NADPH needed for biomass synthesis. Interconversion of NADP to NADH is open for discussion since *B*. *pertussis* only has the NAD(P) transhydrogenase subunit α PntA (BP2897). Subunit β PtnB is a pseudogene. Thus, the enzyme is possibly not active. An NAD^+^ kinase Ppnk (BP2505) may also be involved though a simpler system could be imagined with α-ketoglutarate-glutamate interconversion using the NAD- and NADP-dependent reactions (back and forward reactions) in a futile cycling system. Respiration involving a complete electron transfer chain can provide all the additional ATP, not produced via substrate-level phosphorylation reactions, for growth and O_2_-consumption enabled this to be quantitatively validated.

Thus, *B*. *pertussis* appears to have a fairly simple central metabolic network in which production of biomass, as well as carbon dioxide and ammonium are the only significant outputs, all of these derived from certain amino acids (notably but not exclusively glutamate and proline) and oxygen. When expressed as stoichiometric balance equations, elemental and reducing equivalent recovery is coherent with this situation indicating that a functional TCA cycle is operating. No alternative pathways could be formulated to satisfy the network constraints.

## Discussion

Sequencing analysis, RT-qPCR experiments and enzymatic assays strongly indicate that citrate synthase, aconitase and isocitrate dehydrogenase are functionally produced by *B*. *pertussis* strain Tohama I in the culture conditions used here. This is also confirmed by a carbon stoichiometric flux analysis of *B*. *pertussis* metabolism using the experimental data from the same batch cultures. An entirely functional TCA cycle is coherent with cofactor and energy requirements for the growing microorganism, and with experimental data (O_2_ consumption/CO_2_ production). ATP production appears to be in apparent excess of defined requirements but this has probably been underestimated as ATP essential for amino acid uptake (namely ATP-Binding Cassette (ABC) transport systems) has not been considered here and no idea of maintenance requirements can be estimated from the batch cultures used here. Thus in contrast to a previous report, in which it was claimed that the TCA cycle was not complete, lacking the citrate to α-ketoglutarate segment [10,11], our present results indicate clearly that a functional TCA cycle is operating, as suggested by a recent report optimizing fed-batch substrate feeding requirements [15]. This cyclic organization of acetyl-CoA metabolism is an important contribution to the energetic requirements of the strain. It is however interesting to note that, in our hands, the previously reported accumulation of intracellular end-products (PHB) or exocellular fatty acids, both derived from acetyl-CoA, were not observed. PHB is usually accumulated under conditions of multiple limitations and probably reflects imbalanced growth in the past in which either oxygen transfer was limiting or an extended period of growth was occurring in which key amino acids were already lacking in the medium, thereby blocking growth while other amino acids continued to be consumed. Such intracellular accumulation of PHB is a general manner to temporarily accumulate reserve compounds under conditions in which growth is limiting and is often associated with difficulties in the respiration of the NADH produced in the central pathways. Fatty acids are an alternative overflow solution, though in view of their high demand for NADPH, the energetic determinants are probably rather different. Their exogenous accumulation is toxic for the micro-organism and rarely a significant part of the overall carbon flux. Indeed, Frohlich *et al*. (1995) reported 8.6 ± 3.4 μg/ml of fatty acids at the end of batch cultures but in medium with higher concentrations of glutamate at the onset [14]. Furthermore, they used a different strain of *B*. *pertussis*, derived from the generally atypical 18323 strain, making direct comparison difficult. This does ask the question as to how much variability exists in *B*. *pertussis* strains, though other than loss of certain genome fragments over time, the core genome is highly conserved [9,28]. In light of this, it should be examined why the TCA cycle was initially believed to be non-functional.

Conflicts regarding the TCA cycle in *B*. *pertussis* first arose with cultivation experiments showing that citrate was not a metabolizable substrate for growth [10]. It was therefore assumed that condensation of acetyl-CoA and oxaloacetate by the citrate synthase enzyme was not possible and the hypothesis that genes for the initial reactions of the TCA cycle were probably lacking. In light of the sequenced genomes and the experimental validation of such activities throughout growth, this logic is incorrect though it remains difficult to understand why the bacteria do not metabolize citrate. One possible explanation could have concerned the transport system, assuming that a similar citrate/succinate antiporter is required as described for *E*. *coli* involving the citrate/succinate antiporter CitT (*citT* gene) and the citrate lyase (*citCDEFXG* genes), as well as the two-component system CitA/CitB (*citAB* genes) [29,30]. This two-component system can also be found in other pathogens, such as *Salmonella typhimurium*, *Klebsiella pneumoniae* or *Citrobacter amalonaticus*. The *B*. *pertussis* genome only encodes for CitB and CitE (citrate lyase subunit beta). Perception of extracellular citrate is therefore highly compromised, as well as its transport and metabolism, via this system. However, the lack of this citrate transport system has been overcome by the use of a tripartite tricarboxylate transporter (TTT) involving BctA and BctB as the membrane components and BctC as the periplasmic protein [31]. The operon *bctCBA* is under the control of the signal transduction two-component system encoded by *bctED* and its expression is induced by the presence of citrate in the extracellular medium [31]. The uptake of [1,5-^14^C] citric acid was shown to occur in a *B*. *pertussis* Tohama I derived strain BPSM (streptomycin-resistant) in culture conditions similar to those of the present study [32]. While the BctCBA system has been shown to take up citrate [31], making it difficult to explain the absence of growth on citrate, uptake rates might be too low to sustain an adequate metabolic flux into the TCA cycle in the absence of other carbon sources.

The *B*. *pertussis* strain which was used to propose the incomplete TCA cycle was not the Tohama I strain used in this study, sequenced by Parkhill *et al*. (2003) [9], but strain 509. Therefore, it is possible that the lack of the considered enzyme activities may be a specificity of strain 509. This strain has not been sequenced, though King *et al*. [33] included this strain with others in comparative genomic hybridization (CGH) analysis and showed that the genomic regions that include the citrate synthase (BP2358), aconitase (BP2014 and BP2021) and isocitrate dehydrogenase (BP2488) genes were always present.

Although the experimental evidence presented here seems to be convincing and other configurations of central metabolism, coherent with the experimental data, impossible in the absence of a functional TCA cycle, this could be confirmed with the carbon labeling pattern within TCA cycle intermediates. It could be followed from C13 labeled glutamate though this is not a trivial experiment in view of the loss of certain carbon atoms via the normal transformations which are occurring. In our view, this definitive confirmation is not essential and *B*. *pertussis* should be seen as a bacterium which has a fairly complete metabolic network well suited for growth on the glutamate family of amino acid substrates which enter central metabolism via the TCA cycle.
